# Central nervous system imaging in girls with central precocious puberty: when is necessary?

**DOI:** 10.20945/2359-3997000000259

**Published:** 2020-05-27

**Authors:** Dogus Vuralli, E. Nazli Gonc, Ayfer Alikasifoglu, Nurgun Kandemir, Z. Alev Ozon

**Affiliations:** 1 Hacettepe University Medical School Department of Pediatrics Ankara Turkey Hacettepe University Medical School, Department of Pediatrics, Division of Pediatric Endocrinology, Ankara, Turkey

**Keywords:** Central nervous system, central precocious puberty, idiopathic sexual precocity, magnetic resonance imaging

## Abstract

**Objectives:**

The determinants of an increased risk of an organic pathology underlying central precocious puberty (CPP) in girls remain contentious. The present study aimed to determine the clinical and hormonal findings that can be used to differentiate organic and idiopathic CPP in girls as a screening method so that only those considered likely to have organic CPP undergo cranial magnetic resonance imaging (MRI).

**Subjects and methods:**

The medical records of 286 girls that received GnRH agonist (GnRHa) therapy for CPP were retrospectively evaluated. Chronological and bone age, height, pubertal stage, and basal/stimulated gonadotropin and estradiol (E_2_) levels, as well as cranial MRI findings at the time CPP was diagnosed were recorded. Clinical and hormonal parameters that can be used to differentiate between girls with organic and idiopathic CPP were identified using ROC curves.

**Results:**

Organic CPP was noted in 6.3% of the participants. Puberty started before age 6 years in 88.9% of the girls with organic CPP. Mean E_2_ and peak luteinizing hormone (LH) levels were higher in the girls with organic CPP than in those with idiopathic CPP that were matched for pubertal stage, as follows: early stage puberty (Tanner 2 and 3): E_2_: 62.4 ± 19.8 pg/mL vs. 29.1 ± 9.5 pg/mL; peak LH: 16.8 ± 3.2 IU/L vs. 12.2 ± 3.7 IU/L; advanced stage puberty (Tanner 4): mean E_2_: 87.6 ± 3.4 pg/mL vs. 64.6 ± 21.2 pg/mL; peak LH: 20.8 ± 0.4 IU/L vs. 16.6 ± 5.8 IU/L (P < 0.001 for all). Thresholds for differentiating organic and idiopathic CPP in girls with early-stage puberty were 38.1 pg/mL for E_2_ (100% sensitivity and 80.4% specificity) and 13.6 IU/L for peak LH (100% sensitivity and 66.4% specificity).

**Conclusion:**

Pubertal symptoms and signs generally begin before age 6 years and hormone levels are much higher than expected for pubertal stage in girls with organic CPP. Based on the present findings, cranial MRI is recommended for girls aged < 6 years, as the risk of diagnosing an organic pathology is highest in this age group. Hormone levels higher than expected for pubertal stage might be another indication for cranial MRI, regardless of patient age. Cranial MRI should be performed in girls with early-stage puberty, and an E_2_ level > 38 pg/mL and/or a peak LH level > 13.6 IU/L.

## INTRODUCTION

Organic lesions are present in 5%-10% of girls with central precocious puberty (CPP) ( 1 - 3 ). Cranial magnetic resonance imaging (MRI) can differentiate between organic and idiopathic CPP; however, because idiopathic CPP is more common than organic CPP in girls, it remains unclear if cranial MRI is necessary in all cases. Cranial MRI is recommended in girls with pubertal onset before age 6 years. Neurological findings can suggest an organic cause of CPP, but cranial MRI in girls with pubertal onset after age 6 years is controversial ( 2 - 6 ). Only a few studies on the clinical and hormonal factors that might be predictive of an organic pathology in girls with CPP have been published ( 1 , 7 , 8 ). Young age, rapid progression of puberty, and a high estradiol (E_2_) level are among the factors indicative of organic CPP ( 7 ). The present study aimed to determine the clinical and hormonal findings that can be used to differentiate organic and idiopathic CPP in girls as a screening method so that only those considered likely to have organic CPP undergo cranial MRI.

## SUBJECTS AND METHODS

The medical records of 286 girls that underwent GnRH agonist (GnRHa) therapy due to CPP between 1999 and 2019 were evaluated retrospectively. Chronological age, bone age, height, and pubertal stage, serum follicle stimulating hormone (FSH), serum luteinizing hormone (LH), and serum E_2_ levels, the peak LH level during the GnRH stimulation test, and cranial MRI findings at the time CPP was diagnosed were evaluated. CPP was clinically diagnosed based on breast development (Tanner stage ≥ 2) before age 8 years, and biochemically diagnosed based on a peak LH level ≥ 5 IU/L during the GnRH test ( 9 - 12 ). The GnRH test was performed, as previously described ( 13 ).

Body weight was measured using a digital body-weighing scale, and height was measured in the standing position using a Harpenden stadiometer. The height standard deviation scores (SDSs) for chronological age and bone age were calculated. Tanner staging was used to determine pubertal stage ( 14 ). Bone age was determined by a pediatric endocrinologist, according to Greulich and Pyle ( 15 ).

Commercial immunochemiluminometric assay (ICMA) kits were used to measure FSH, LH, and E_2_ (ARCHITECT System, Abbott Laboratory Diagnostics, USA) levels. Detection limits, and intra- and inter-assay coefficients of variation were, respectively, 0.07 IU/L, 1.7%-3.1%, and 2.4%-3.9% for LH, 0.3 IU/L, 2.8%-4.2%, and 3.3%-4.6% for FSH, and 10 pg/mL, 1.8%-7.4%, and 1.7%-6.4% for E_2_.

All the girls underwent cranial/pituitary MRI with contrast (gadolinium) material. Cases with cranial/pituitary pathology were diagnosed as organic CPP. Incidentalomas are lesions detected when imaging methods are performed for other reasons, rather than for identifying an excess or lack of secretion of pituitary hormones ( 16 ). Girls with incidentalomas were considered as idiopathic CPP. The best threshold values for basal E_2_ and peak stimulated LH for differentiating organic and idiopathic CPP were identified using ROC curves.

### Statistical analysis

Statistical analysis was performed using IBM SPSS Statistics for Windows v.19.0 (IBM Corp., Armonk, NY, USA). Continuous variables are shown as mean ± SD, and categorical variables are shown as number and percentage. For analysis of differences between independent groups Student’s t-test was used for normally distributed data, whereas the Mann-Whitney U test was for data not normally distributed. Categorical variables were analyzed using the non-parametric chi-square test. ROC curves were used to identify the factors that best differentiated girls with organic and idiopathic CPP. The level of statistical significance was set at P < 0.05.

## RESULTS

Organic CPP was diagnosed in 6.3% (n = 18) of the 286 participants. In all, 11 of the 18 girls with organic CPP were neurologically symptomatic and had previously known CNS pathologies. Among the 11 girls that were neurologically symptomatic at the time CPP was diagnosed, 9 were previously followed-up for mental motor retardation and epilepsy, of which 3 had a developmental anomaly of the CNS, 3 had a parenchymal injury based on cranial MRI, and 3 had hydrocephalus. These 9 girls were aged 2-6 years at the time CPP was diagnosed. Among the other 2 girls with organic CPP that were neurologically symptomatic, 1 had neurofibromatosis type 1, with characteristic cafe-au-lait spots and axillary freckles that were noted during physical examination, as well as an optic pathway glioma based on cranial MRI. This patient had loss of visual acuity due to the tumor in the optic pathway and had initiation of pubertal symptoms at age 6.5 years. The other girl had clinical such findings as impaired vision, developmental delay, intellectual disability, and cranial MRI findings of optic nerve hypoplasia, as well as agenesis of the septum pellucidum and corpus callosum. In addition, she was diagnosed as septo-optic dysplasia (SOD) and initiation of her pubertal symptoms began at age 6.2 years.

The remaining 7 girls with organic CPP were neurologically asymptomatic at the time CPP was diagnosed. Organic lesions were detected in these 7 girls, as follows: hypothalamic hamartoma (n = 2); suprasellar arachnoid cyst (n = 2); macroadenoma (n = 2); optic glioma (n = 1) ( Table 1 ). The 2 patients with a hypothalamic hamartoma were diagnosed as CPP before age 2 years, whereas the other 5 girls with space-occupying lesions were diagnosed as CPP between 2 and 6 years of age. The suprasellar arachnoid cysts noted in 2 girls were > 5 cm in diameter and were causing hydrocephalus, and papilledema was observed via neuro-ophthalmological examination. No other neuro-ophthalmological signs or symptoms were present in any of the remaining patients with space-occupying lesions.

**Table 1:** Age of onset of pubertal findings in girls with organic and idiopathic CPP

	Age at onset of pubertal findings		
	0-2 yrs	2-6 yrs	6-8 yrs
	n (%)	n (%)	n (%)
Idiopathic CPP		36 (72)	232 (99.1)
		(4 incidentalomas: microadenoma and para intermedia cysts)	(18 incidentalomas: microadenoma and para intermedia cysts)
Organic CPP	2 (100)	14 (28)	2 (0.9)
	2 Hypothalamic hamartomas	2 suprasellar arachnoid cysts > 5cmin size and causing hydrocephalus 2 hemorrhagic macroadenomas 1 optic glioma 3 developmental anomaly of CNS 3 hydrocephalus 3 parenchymal injury, necrotic lesions	1 optic glioma (NF 1) 1 septo-optic dysplasia
Total	2 (100)	50 (100)	234 (100)

In all, 22 incidentalomas, including microadenomas and millimetric pars intermedia cysts, were noted in the 268 girls with idiopathic CPP ( Table 1 ). In total, 4 of the 22 incidentalomas were diagnosed at age 2-6 years, whereas 18 of the 22 were diagnosed at age 6-8 years. In 88.9 % (16/18) of the girls with organic CPP pubertal findings began before age 6 years ( Table 1 ).

CPP was diagnosed at an earlier age, bone age was more advanced, the bone age-corrected height-SDS was lower, and sex steroid levels and the peak LH level (GnRH test) were higher in the girls with organic CPP than in those with idiopathic CPP ( Table 2 ). Breast development at the time CPP was diagnosed was Tanner stage 2 (T2) in 35.3% (n = 101) of the 286 participants, T3 in 52.4% (n = 150), and T4 in 12.2% (n = 35). Pubic hair at the time CPP was diagnosed was T2 in 39.2% (n = 112) of the 286 participants, T3 in 51% (n = 146), and T4 in 9.8% (n = 28). Pubertal stage in the girls with idiopathic and organic CPP did not differ significantly ( Table 2 ). None of the 286 girls had menarche prior to GnRHa treatment. In all, 53 of the 268 idiopathic CPP cases had a family history of early puberty ( Table 2 ). Family history of early puberty was paternal in 16 whereas it was on the maternal side in 26 of 53 girls. In 11 girls both maternal and paternal side had history of early puberty.

**Table 2:** Clinical and hormonal characteristics in the patients with organic and idiopathic CPP

	Girls		

	Organic (n = 18)	Idiopathic (n = 268)	P value
Chronological age at diagnosis (CA) (yrs)	4.7 ± 1.2	7.9 ± 0.9	< 0.001
Age at initiation of symptoms (yrs)	4.0 ± 1.2	6.9 ± 0.9	< 0.001
Bone age (BA) (yrs)	8.3 ± 1.1	10.1 ± 0.8	< 0.001
BA advancement (BA-CA) (yrs)	3.6 ± 1.2	2.2 ± 0.9	< 0.001
Height-SDS	1.8 ± 1.0	1.5 ± 0.8	0.384
Height-SDS for BA	-2.3 ± 0.8	-0.7 ± 0.7	< 0.001
Pubertal stage (Breast development)			
T2	6 (33.3%)	95 (35.5%)	
T3	10 (55.6%)	140 (52.2%)	
T4	2 (11.1%)	33 (12.3%)	
Basal FSH (IU/L)	3.7 ± 1.1	4.3 ± 1.6	0.525
Basal LH (IU/L)	1.5 ± 0.9	1.4 ± 1.0	0.422
Basal E_2_ (pg/mL)	65.2 ± 22.4	28.6 ± 14.6	< 0.001
Peak stimulated LH (IU/L)	17.2 ± 3.6	12.7 ± 4.3	< 0.001
Family history of early puberty	0	53 (18.5%)	< 0.001

Comparison of hormone levels according to pubertal stage showed that the basal E_2_ and peak stimulated LH levels were higher in the girls with organic CPP than in those with idiopathic CPP ( Table 3 ). Basal E_2_ and peak LH levels in the girls with organic CPP and early-stage puberty (Tanner stage 2 and 3) were similar to those in the girls with idiopathic CPP and advanced-stage puberty (Tanner stage 4) (basal E_2_: 62.4 ± 19.8 pg/mL vs. 64.6 ± 21.2 pg/mL, P = 0.844; peak LH: 16.8 ± 3.2 IU/L vs. 16.6 ± 5.8 IU/L, P = 0.642).

**Table 3:** Hormone levels according to pubertal stage in the girls with organic and idiopathic CPP

	Early pubertal stages (T2&T3)			Advanced pubertal stages (T4)		
Hormone levels	Idiopathic (n: 235)	Organic (n: 16)	P value	Idiopathic (n: 33)	Organic (n: 2)	P value
Basal E_2_ (pg/ml)	29.1 ± 9.5	62.4 ± 19.8	<0.001	64.6 ± 17.6	87.6 ± 4.8	< 0.001
Peak stimulated LH (IU/L)	12.2 ± 3.7	16.8 ± 3.2	<0.001	16.6 ± 4.8	20.8 ± 0.6	< 0.001

The best cut-off points for differentiating girls with organic CPP and idiopathic CPP with early-stage puberty (Tanner stage 2 and 3) were basal E_2_ of 38.1 pg/mL (100% sensitivity, 80.4% specificity) and peak LH of 13.6 IU/L(100% sensitivity and 66.4% specificity) ( Table 4 ). Significant cut-off points for basal E_2_ and peak stimulated LH that could be used to differentiate girls with organic and idiopathic CPP and advanced-stage puberty were not observed.

**Table 4:** The best threshold values for differentiating organic and idiopathic CPP

	Basal E_2_ levels					Peak stimulated LH				
	
	Cut-off (pg/ml)	Sensitivity (%)	Specifity (%)	AUC	p	Cut-off (IU/L)	Sensitivity (%)	Specifity (%)	AUC	p
Organic vs idiopathic CPP (early stages- T2&T3)	38.1	100	80.4	0.945	<0.001	13.6 15.4 19.1	100 66.7 33.3	66.4 80.0 100	0.844	<0.001

## DISCUSSION

In the present study organic pathology was detected in approximately 1 of every 3 girls with CPP aged < 6 years, whereas only 2 of 234 girls with CPP aged > 6 years had an organic pathology. A few recent studies that classified girls with CPP according to age at diagnosis as < 6 years and ≥ 6 years reported that 17.1%-26.9% of girls diagnosed at age < 6 years had a CNS pathology, *versus* 0%-1.9% of those diagnosed at age ≥ 6 years ( 2 , 8 , 17 ). Based on the present findings, with a high probability of a CNS lesion in girls aged < 6 years, we recommend cranial MRI, as reported earlier ( 18 ).

The use of cranial MRI in girls with CPP and pubertal onset at age 6-8 years is a contentious issue ( 2 - 6 , 18 , 19 ). Some studies suggest that routine cranial MRI should not be performed in girls with pubertal onset at age > 6 years if they have normal neurological findings ( 2 , 4 ); however, others report that MRI should be performed in all girls, regardless of age, as a routine evaluation for the possibility of an underlying CNS lesion, however small it may be ( 3 , 5 , 6 ). Other studies suggest that clinical and hormonal parameters lack the sensitivity and specificity to predict an organic CNS lesion; thus, cranial MRI should be performed in all girls diagnosed with CPP, regardless of age of onset ( 1 ). One of the constraints of these studies is that they included small patient groups, in contrast to the present study, which included a cohort of 286 girls with CPP. Cranial MRI is expensive, and can sometimes require sedation and intravenous gadolinium injection; therefore, it is useful to differentiate girls at a high-risk for organic CPP, which will require additional clinical studies.

Several studies analyzed various clinical and biochemical factors that might predict intracranial pathology in girls with CPP. Although organic causes are rare in girls, some clinical and laboratory findings point to an organic etiology for CPP. Clinically, the probability of a CNS pathology is higher in girls aged < 5 years that have rapid progression of puberty and significant bone age advancement ( 5 , 20 , 21 ). Data from studies that compared idiopathic and organic CPP published inconsistent findings. Some studies noted higher basal gonadotropin levels, stimulated LH and FSH peaks, and basal serum E_2_ levels in girls with organic CPP, whereas others did not ( 6 , 17 , 19 , 22 - 25 ). In the present study the girls with organic CPP had earlier age of onset with advanced bone age and higher sex steroid levels, as well as higher peak stimulated LH levels than those with idiopathic CPP.

Among girls in the present study with a similar pubertal stage, basal E_2_ and peak stimulated LH levels were significantly higher in those with organic CPP, as compared to those with idiopathic CPP; therefore, we recommend cranial MRI in such cases, as the possibility of an underlying organic lesion increases hormone levels higher than expected for pubertal stage are observed.

Chalumeau and cols. designed an evidence-based diagnosis tree that can predict a CNS pathology in girls with CPP ( 17 ). They showed that CPP onset before age 6 years, lack of pubic hair, and a basal estrogen level > 45 pmol/L were the most important parameters that predict organic CPP. In the present study a basal serum E_2_ level > 38 pg/mL and a peak stimulated LH level > 13.6 IU/L were the best parameters for differentiating organic and idiopathic CPP in girls with early-stage puberty (Tanner stage 2 and 3), regardless of age. As the present study included a small number of girls with advanced-stage puberty, significant cut-off points for differentiating organic CPP could not be observed.

Some cases of CPP are familial; also monogenic forms of CPP are identified. Mutations in four genes have been associated with monogenic forms of CPP. Monoallelic gain-of-function mutations in the genes *KISS1* (encoding kisspeptin) and *KISS1R* (encoding kisspeptin receptor) were shown in some cases with familial CPP suggesting an autosomal dominant inheritance ( 26 - 28 ). Another important cause of familial CPP is loss-of-function mutations in *MKRN3* (encoding makorin ring finger protein 3) ( 29 ). *MKRN3* , is an imprinted gene (maternal silencing), only the paternal gene copy is expressed. Thus, there is paternal inheritance with an autosomal dominant pattern. Delta-like homolog 1 ( *DLK1* ) is another paternally expressed imprinted gene that has a role in CPP.

Until now most of the monogenic cases involved *MKRN3* mutations, whereas other mutations appear to account for very few cases ( 28 , 30 ). A study that included 40 individuals from 15 families reported that 15 children and 5 families had *MKRN3* mutations ( 29 ). All cases with *MKRN3* mutations had normal cranial MRI. Latronico and cols. recommend that patients with a positive family history of CPP suggesting paternal inheritance may be analyzed for *MKRN3* mutations instead of cranial MRI ( 7 ). 10% of the girls in the current study had a family history of paternal side. However, it was not possible to perform genetic testing in those patients.

### Study limitations

Although the present study is among the largest in the literature, in terms of the total number of cases and the number of cases with organic CPP, the number of organic CPP cases with advanced-stage puberty was very low, which is considered a limitation.

In conclusion, pubertal symptoms in girls with organic CPP generally begin before age 6 years. In addition, hormone levels are much higher than expected for pubertal stage in girls with organic CPP. Accordingly, cranial MRI should be performed in all girls aged < 6 years of age, as well as in those with hormone levels higher than expected for pubertal stage, regardless of age. Based on the present findings, we do not suggest routinely performing cranial MRI in girls aged > 6 years, unless they exhibit signs of organic lesions, such as neurological findings or hormone levels higher than expected for pubertal stage. In contrast, cranial MRI should be performed in girls with early-stage puberty (Tanner stage 2 and 3), and an E_2_ level >38 pg/mL and/or a peak LH level > 13.6 IU/L.
